# Brief report on the relation between complement C3a and anti dsDNA antibody in systemic lupus erythematosus

**DOI:** 10.1038/s41598-022-10936-z

**Published:** 2022-05-02

**Authors:** Yao-Hua Cai, Jun Deng, Zhao-Lin Chen, Heng Mei, Liang Tang, Shan-Shan Luo, Yu Hu

**Affiliations:** 1 Institute of Hematology, Union Hospital, Tongji Medical College, Huazhong University of Science and Technology, No. 1277 Jiefang Avenue, Wuhan, 430022 Hubei China; 2 Hubei Clinical and Research Center of Thrombosis and Hemostasis, Wuhan, Hubei China; 3 Institute of Hematology, The Second Affiliated Hospital of Guangzhou Medical University, Guangzhou, Guangdong China

**Keywords:** Predictive markers, Autoimmunity, Complement cascade, Rheumatic diseases

## Abstract

Systemic lupus erythematosus (SLE) is a complex autoimmune disease characterized by the production of a diverse array of autoantibodies and the dysfunctional activation of the complement system. The specific association between the complement component C3a (C3a) protein and antibodies specific for double-stranded DNA (anti-dsDNA), however, has not been studied in detail to date. This study was thus designed to more fully explore circulating C3a levels in SLE patients. In total, 13 SLE patients were enrolled in this study after having been diagnosed in accordance with the SLICC classification criteria, with 7 and 6 patients respectively exhibiting positivity for anti-dsDNA and anti-Sm autoantibodies. Serum complement component C1q (C1q) and C3a levels in samples from these patients were detected via Western blotting, while other serological, biochemical, and clinical parkers associated with disease activity were detected using standard laboratory techniques. The levels of serum C3a in anti-dsDNA+ patients were significantly elevated as compared to those in anti-Sm+ patients (*P* < 0.01), and a positive correlation between serum C3a levels and SLE Disease Activity Index scores was detected (*P* < 0.05, *r* = 0.6134). C3a levels are correlated with the degree of SLE disease activity and other clinically relevant readouts in SLE patients. C3a levels may also enable the differentiation between inactive and active SLE, while also offering value as an advantageous biomarker for thrombophilia monitoring in SLE patients.

## Introduction

The complement system is an evolutionarily ancient facet of the innate immune system that plays an integral role in protecting the host against many potential pathogens^1^. Different stimuli can induce complement activation through three different pathways, ultimately resulting in a shared response conducive to the elimination of infection and the restoration of immune homeostasis^2,3^. The small anaphylatoxin subunits of the C3 and C5 complement proteins (C3a and C5a) are important drivers of inflammatory responses, promoting mast cell degranulation, smooth muscle contraction, enhanced vascular permeability, immune cell chemotaxis, and proinflammatory cytokine release^4^. While complement is beneficial when appropriately activated, its dysfunctional or inappropriate activation can contribute to a loss of host homeostasis that can facilitate infection, autoimmunity, oncogenic progression, and/or tissue damage^2^.

Systemic lupus erythematosus (SLE) is a persistent, debilitating autoimmune disease that primarily affects women and can occur in individuals of any age, sex, or ethnicity^5^. SLE patients commonly exhibit a wide range of autoantibodies and dysfunction of the complement system that can contribute to the incidence of tissue and organ damage^6^. The most common autoantibodies unique to SLE patients are those specific for Smith (Sm) and double-stranded DNA (dsDNA), with the presence of one or more of these autoantibodies being important to SLE disease classification^7^. The levels of anti-dsDNA often vary over time in SLE patients, and in some cases may even disappear with appropriate treatment^8^. Anti-Sm Ab levels, in contrast, tend to be stable and resistant to treatment-related changes^7^.

As the activation of the complement system occurs in SLE patients during disease flares, complement protein production, activation, and consumption are thought to be relatively proportional to the degree of disease activity. The anaphylatoxin C3a is a small molecular byproduct of complement activation and one of the primary pro-inflammatory components of this system, yet its value as a predictor of SLE patient acute disease status is relatively poorly understood at present. Both C3a and anti-dsDNA levels can be indicative of an acute phase response, but the precise relationship between these two serum biomarkers in SLE patients is incompletely understood. Here, we therefore explored the relationship between serum C3a and anti-dsDNA levels, D-dimer levels, and SLE Disease Activity Index (SLEDAI) scores in a small patient cohort. This analysis revealed a significant correlation between the C3a and both anti-dsDNA levels and SLEDAI scores in this patient cohort, highlighting the promise of C3a as a clinical biomarker that can be used to monitor disease progression and evaluate patient prognosis.

## Patients and study design

### Study participants

Samples of blood were obtained from 13 consecutive SLE patients identified in accordance with the Systemic Lupus International Collaborative Clinics (SLICC) classification criteria^9^, including 6 and 7 patients respectively positive for anti-Sm and anti-dsDNA autoantibodies (46.2% and 53.8%, respectively; Table 1). Healthy donor blood was also collected. All blood samples were collected within 24 h following admission and prior to treatment. Whole blood was allowed to clot for 30 min at room temperature and on ice for 1 h, after which samples were centrifuged for 10 min at 4 °C. Serum was then collected from the supernatant and stored at − 80 °C prior to analysis.

**Table 1:** Clinical, biochemical, and laboratory data for the 13 enrolled SLE patients.

Patients code	AS-1	AS-2	AS-3	AS-4	AS-5	AS-6	AD-1	AD-2	AD-3	AD-4	AD-5	AD-6	AD-7
Age, years	57	30	33	50	44	23	64	53	16	32	48	68	44
Disease duration, years	13	2	0	10	3	0	18	1	2	1	0	14	0
SLEDAI score	6	7	6	7	8	2	9	8	4	13	22	13	23
ESR (mm/h)	13	12	14	10	2	7	8	9	19	9	4	7	120
Albumin (g/dl)	35.5	42	37.6	31.8	44.8	40	34.4	36.7	35.7	34.6	23.2	36.6	28.1
CK (U/L)	–	51	33	27	34	11	66	36.7	27	16	9	25	12
LDH (U/L)	–	369	180	225	187	169	251		273	191	168	201	280
α-HBDH (U/L)	–	267	130	164		168	205		191	144	131	156	216
IL-2	0.79	4.21	0.89	0.79	2.03	2.19	1.29	0.76	1.29	–	0.97	–	11.31
IL-4	1.45	2.34	1.41	1.27	2.49	2.52	1.87	1.69	0.94	–	1.39	–	3.43
IL-6	14.01	10.73	3.79	1.86	4.16	5.63	25.99	10.56	27.17	–	196.17	–	29.33
IL-10	2.34	2.32	2.18	1.69	3.73	4.29	2.68	3.36	6.01	–	5.73	–	9.3
TNF-a	17.83	2.06	1.37	1.55	2.71	12.7	10.8	10	2.08	–	49.33	–	8.04
IFN-y	1.7	1.46	1.18	1.49	2.32	2.64	1.54	1.41	2.1	–	1.07	–	39.71
CD4/CD8	1.17	0.3	2.05	0.29	0.96	0.65	1.24	0.87	1	–	0.89	0.5	2.1
anti-Sm Ab (AI)	> 8.0	1.9	5.9	1.9	2.9	1.4	< 0.2	0.3	< 0.2	< 0.2	< 0.2	< 0.2	0.3
anti-ds DNA Ab (IU/ml)	< 1	1	< 1	2	1	7	84	58	> 300	29	32	17	> 300
IgE (g/L)	11.7	10.62	62.9	10.85	44.45	81.98	67.81	44.99	36.92	189.9	38.87	66.07	21.51
IgG (g/L)	8.24	11.5	15.9	16	10.5	9.42	8.74	11	14.8	14	6.54	13.9	9.54
IgA (g/L)	1.06	1.39	2.9	0.69	2.81	1.47	4.25	2.12	3.11	1.98	0.88	3.21	2.45
IgM (g/L)	0.687	0.649	1.24	0.252	0.449	0.718	1.01	0.468	1.01	0.949	0.285	0.701	0.888
C3 (g/L)	0.635	0.962	0.77	1.03	0.767	0.524	0.599	0.555	0.54	0.26	0.426	0.536	0.427
C4 (g/L)	0.16	0.256	0.226	0.24	0.169	0.147	0.12	0.183	0.085	0.1	0.13	0.125	0.052
CRP (mg/L)	< 2.98	13.2	< 2.98	21.9	< 2.98	< 2.98	< 2.98	< 2.98	7.37	< 2.98	< 2.98	< 2.98	5.6
PCT (ug/L)	< 0.13	< 0.13	< 0.13	0.31	< 0.13	–	–	< 0.13	0.24	–	–	–	< 0.13
D-D (mg/L)	0.49	0.22	0.57	1.04	0.27	0.22	1.74	1.50	1.43	0.31	1.06	0.33	1.09
FDP (ug/ml)	4	4	4	4	1	1	5.9	–	4	4	4	1	2
ATIII (%)	98	119	92	84	97	98	92	–	94	98	84	86	99
PT (s)	12.7	12.7	12.9	13.6	12.7	12.8	12.7	–	13.3	11.9	14.3	13.1	13.4
APTT (s)	34	34.9	34.1	39.2	33.7	40	31	–	38.3	33.3	84	38.9	28.1
FIB (g/L)	3.51	3.98	2.34	5.1	3.36	2.32	2.09	–	3.18	3.28	1.75	2.8	2.89
TT (s)	15.8	15.2	17.4	17	16.7	16.2	15.6	–	15.7	15.9	16.2	16.7	16.7

*AS* anti-Sm antibodies (+), *AD* anti-dsDNA antibodies (+), *U-LEU* urinary leukocyte, *FDP* fibrin degradation product, *ATIII* Antithrombin III, *ESR* Erythrocyte sedimentation rate, *CRP* C-reactive protein, *PCT* procalcitonin, *PT* prothrombin time, *APTT* activated partial thromboplastin time, *FIB* blood fibrinogen, *CK* creatine kinase, *LDH* lactate dehydrogenase, *α-HBDH* alpha-hydroxybutyrate dehydrogenase, *PLT* platelet, *WBC* white blood cell.

All participants provided written informed consent to participate, and the Ethics Committee of Union Hospital at Huazhong University of Science and Technology approved this study, which was performed according to approved guidelines. The Systemic Lupus Erythematosus Disease Activity Index-2000 (SLEDAI-2K) was used to measure SLE disease severity^10^.

### Standard laboratory investigations

Standard laboratory-based coagulation tests (including D-Dimer, fibrin degradation product (FDP), and Antithrombin III (ATIII)), inflammatory marker tests (including Erythrocyte sedimentation rate (ESR), C-reactive protein (CRP), and procalcitonin (PCT)), and liver/kidney functional assays were conducted. Standard approaches were used to detect cytokines (Interleukin(IL)-2, IL-4, IL-6, IL-10), autoantibodies, and complement (C3 and C4) proteins.

### Complement protein measurement

Levels of C1q and C3a in patient serum were detected via Western blotting. Serum samples (0.5 µL) were initially combined with 7.5 µL of PBS and 2 µL of rot-load 1 for 10 min at 98 °C, after which samples were separated via 12% SDS-PAGE and transferred to PVDF membranes. Blots were subsequently blocked overnight using 4% skimmed milk and 1% BSA (containing 0.05% Tween-20) at 4℃, followed by incubation for 1 h Rabbit anti-human C3a (Cat No. #A218,.) or Goat anti-human C1q (Cat No. #A200) (Both diluted 1:2000; Complement Technology, Inc.). Blots were then washed, probed with secondary HRP-conjugated goat anti-rabbit IgG (1:2000; Cat No. SA00001-2, Proteintech, Inc.) and rabbit anti-goat IgG (1:2000; Cat No. SA00001-4, Proteintech, Inc.), and protein bands were then detected with a ChemiDoc™ XRS+ Imaging System and the Image Lab™ Software (v 5.2). (Bio-Rad Laboratories, Inc.). C3a levels were also quantified via ELISA (Cat No. E-EL-H0818c, Elabscience, Inc.).

### Statistical analyses

GraphPad Prism (v 5.01) was used for all data analyses. The obtained data passed Normality and homogeneity test prior to the statistical analysis. Mean serum C1q and C3a levels were assessed via Student’s t-tests. Correlations between C3a levels and other parameters of interest including SLEDAI scores were made using Pearson correlation tests. Data are presented as mean with standard deviation (SD), and a two-tailed *P* < 0.05 was the threshold of significance.

## Results

### SLE patient clinical characteristics

Clinical characteristics were initially compared between SLE patients exhibiting positivity for anti-Sm and anti-dsDNA autoantibodies (Table 2). In total, 13 patients were enrolled in this study, with respective age values(mean ± SD) in the anti-Sm+ and anti-dsDNA+ cohorts of 39.5 ± 13.0 and 46.4 ± 18.1 years, and respective disease duration values(mean ± SD) of 4.67 ± 5.50 and 5.14 ± 7.54 years. There were no significant differences in demographics, laboratory findings, or SLEDAI scores among groups.

**Table 2:** SLE patient clinical characteristics.

Features	SLE	SLE
	anti-Sm Ab (+)	anti-dsDNA Ab (+)
Age, years, mean ± SD	39.5 ± 13.0	46.4 ± 18.1
Sex, women/men	5/1	6/1
Disease duration, years, mean ± SD	4.67 ± 5.50	5.14 ± 7.54
SLEDAI score, mean ± SD	6 ± 2.10	13.14 ± 7.10
Low C3/C4 (yes/no)	4/2	7/0
Fever (yes/no)	2/4	1/6
Lupus rash (yes/no)	2/4	0/7
Alopecia (yes/no)	1/5	1/6
Mucosal ulcers (yes/no)	0/6	2/5
Arthritis (yes/no)	0/6	2/5
Myositis (yes/no)	2/4	1/6
Psychosis (yes/no)	1/5	0/7
Organic brain syndrome (yes/no)	1/5	0/7
Cranial nerves disorder (yes/no)	1/5	0/7
Vasculitis (yes/no)	0/6	2/5
Pericarditis (yes/no)	1/5	0/7
Active urinary sediment (yes/no)	0/6	1/6
Hematuria (yes/no)	0/6	3/4
Proteinuria (yes/no)	1/5	5/2
Leukocyturia (yes/no)	0/6	1/6
Leukopenia (yes/no)	1/5	4/3

*anti-Sm Ab* anti-Smith antibodies, *anti-dsDNA* anti-double-stranded DNA antibodies, *SLEDAI* Systemic Lupus Erythematosus Disease Activity Index.

### SLE patients positive for anti-dsDNA exhibit higher serum C3a levels

**Figure 1:**
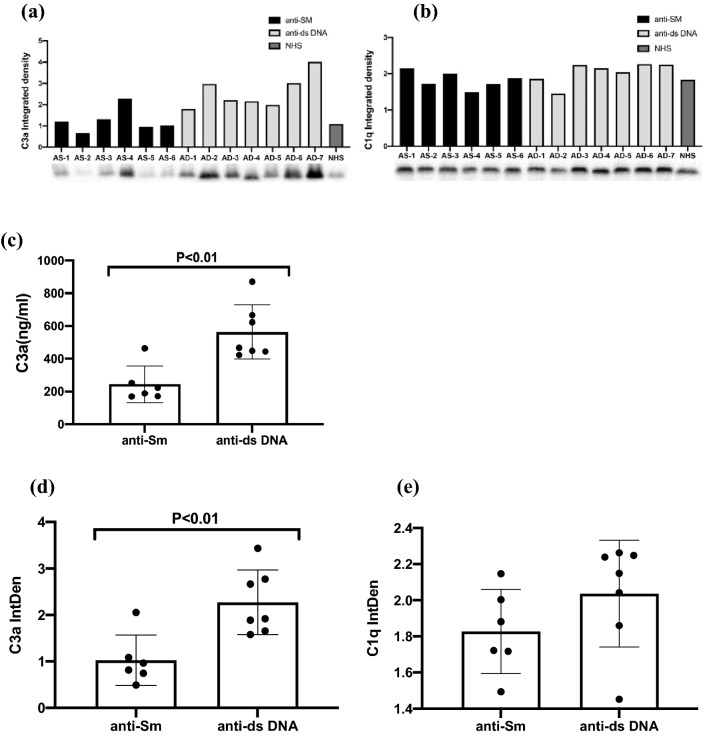
Serum C3a levels in SLE patients positive for anti-Sm and anti-dsDNA autoantibodies. (**a**,**b**) 6 SLE patients positive for anti-Sm autoantibodies, and 7 SLE patients positive for anti-dsDNA autoantibodies, C3a and Cq1 levels were measured via Western blotting. (**c**) Serum samples from donors collected as in (**a**) were used to assess C3a levels via ELISA based on provided directions. (**d**) C3a levels were significantly higher in SLE patients positive for anti-dsDNA as compared to anti-Sm+ patients (*P* < 0.01). (**e**) No significant differences in serum C1q levels were observed between groups. Data were compared via Student’s t-tests, with dots corresponding to individual samples.

Western blotting was next used to assess serum C1q and C3a levels in SLE patients positive for anti-SM or anti-dsDNA antibodies (n = 6 and n = 7, respectively) (Fig. 1a,b), with ELISAs similarly being conducted (Fig. 1c). Anti-dsDNA+ patients exhibited significantly higher serum C3a integrated density values (mean = 2.59, SD = 0.79) relative to anti-SM+ patients (mean = 1.24, SD = 0.561) (Fig. 1d; *P* < 0.01; n = 13), whereas there were no differneces in serum C1q levels between groups (Fig. 1e; *P* = 0.1812; n = 13), consistent with a close relationship between anti-dsDNA and the overactivation of the complement system.

### Serum C3a levels are positively correlated with SLE disease severity

**Figure 2:**
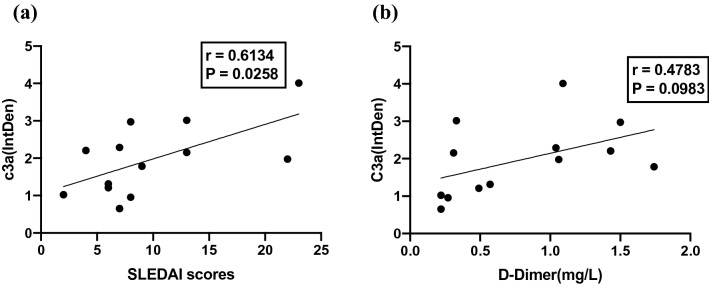
Correlations between serum C3a, SLEDAI scores, and D-dimer levels. (**a**) Serum C3a levels were significantly positively correlated with SLEDAI scores. (**b**) Serum C3a levels exhibited a trend towards correlation with D-dimer levels in SLE patients. *P* < 0.05 was the threshold of significance. SLEDAI: Systemic Lupus Erythematosus Disease Activity Index.

Spearman rank correlations were next used to examine the relationship between serum C3a levels and SLEDAI scores (Fig. 2a), revealing a significant positive correlation between these variables (*P* < 0.05, *r* = 0.6134). C3a levels were also somewhat positively correlated with D-dimer levels, but this relationship was not significant (Fig. 2b; *P* = 0.0983, *r* = 0.4783). These results thus suggest that C3a levels may offer value as a predictor of disease severity in patients with SLE.

## Discussion

SLE is a complex, inflammatory, progressive autoimmune condition characterized by high rates of recurrence and an inconsistent disease course^11^. The complement system plays an essential role in central innate immune surveillance, and can be activated via the classical, alternative, and lectin pathways. Dysfunctional complement activity can contribute to the pathogenesis of SLE and many other conditions including Disseminated intravascular coagulation (DIC), Paroxysmal nocturnal hemoglobinuria (PNH), and Atypical hemolytic uremic syndrome (aHUS)^12^. At present, C3 and C4 levels are commonly used to monitor disease activity in patients with SLE, but there are many limitations to their utility given that these proteins are generally only detected at low levels and are rare in individuals with mild or early-stage disease^13^.

Here, we detected increased serum C3a levels in SLE patients positive for anti-dsDNA but not anti-Sm autoantibodies, and we further observed a positive correlation between C3a levels and SLEDAI scores. We analyzed the C3 and C3a level of different patient samples using C3 and C3a ELISA kit, and compared with each other. The data showed that the correlation of C3 with disease activity matches to some extend (supplementary information Fig. 1a). As the split products of C3, increased C3a is associated with reduced C3 (supplementary information Fig. 1b). However, the *r* value is kindly low. The reason might be that C3a are degraded to some extend upon the long storage time. As we know, the small cleavage product C3a is quite unstable, while C3 is kindly stable. Fragmentary C3a and C5a are rapidly produced following complement cascade activation, thus providing a more reliable readout for the activation of this system relative to intact C3 or C5 protein levels^14^. Moreover we found Anti-dsDNA+ patients are more likely to have nephritis symptom, perhaps because signaling of the C3a anaphylatoxin through its G protein-coupled receptor(C3aR), contribute to lupus nephritis^15^.Consistent with our observed correlation between C3a levels and SLE patient disease activity, prior studies have reported increases in complement split products and disease severity, although the specific association between such activity and C3a has not been reported owing to the very short half-life exhibited by C3a in serum^16^.

We additionally observed a trend towards a relationship between serum C3a and D-dimer levels in SLE patients. The anaphylatoxins C3a and C5a have been reported to promote neutrophil and macrophage activation, resulting in the increased release of vasoactive compounds conducive to increased vascular inflammatory activity. While healthy individuals exhibit low C3a and C5a levels, they increase rapidly in the context of complement overactivation, suggesting that the formation of immune complexes, inappropriate complement activation, and consequent damage to the vascular endothelium may contribute to vasculopathy observed in patients with SLE. Overall, these results support the potential value of C3a as a biomarker that can be used to monitor disease activity in patients suffering from SLE and thrombophilia.

There are certain limitations to this analysis. For one, this was a relatively small study of 13 patients and the results are potentially susceptible to selection bias, with the limited sample size further restricting additional analyses of C3a levels, further constraining these findings. Additional large-scale multi-center studies will thus be vital to explore the mechanistic relationship between SLE and complement levels. In order to avoid treatment-related effects on complement levels, we restricted the present study to patients for whom blood samples were obtained within 24 h of admission and prior to treatment, thus partially accounting for our small sample size.

As C3a can be readily degraded and necessitates specialized sample handling, its clinical utility is potentially limited. However, we herein found that the data generated from a C3a ELISA kit were reliable and thus potentially amenable to clinical use, if one avoid long storage and freeze and thaw cycles. The use of C3a as a biomarker to gauge SLE patient disease activity may further be constrained by the fact that not all increases in C3a levels are tied to SLE disease flares given that other stimuli such as tumors, pathogens, or other autoimmune diseases can also induce excessive complement system activation. The precise explanation for the observed positive correlation between anti-dsDNA levels and complement overactivation is also incompletely understood at present, highlighting a promising direction for further research.

## Supplementary Information

Supplementary Information 1.

Supplementary Information 2.

Supplementary Information 3.
