# Supplementary figures and images for: Brief report on the relation between complement C3a and anti dsDNA antibody in systemic lupus erythematosus
Source: Sci Rep. 2022 May 2;12:7098. doi: 10.1038/s41598-022-10936-z (PMC9061720; doi:10.1038/s41598-022-10936-z)

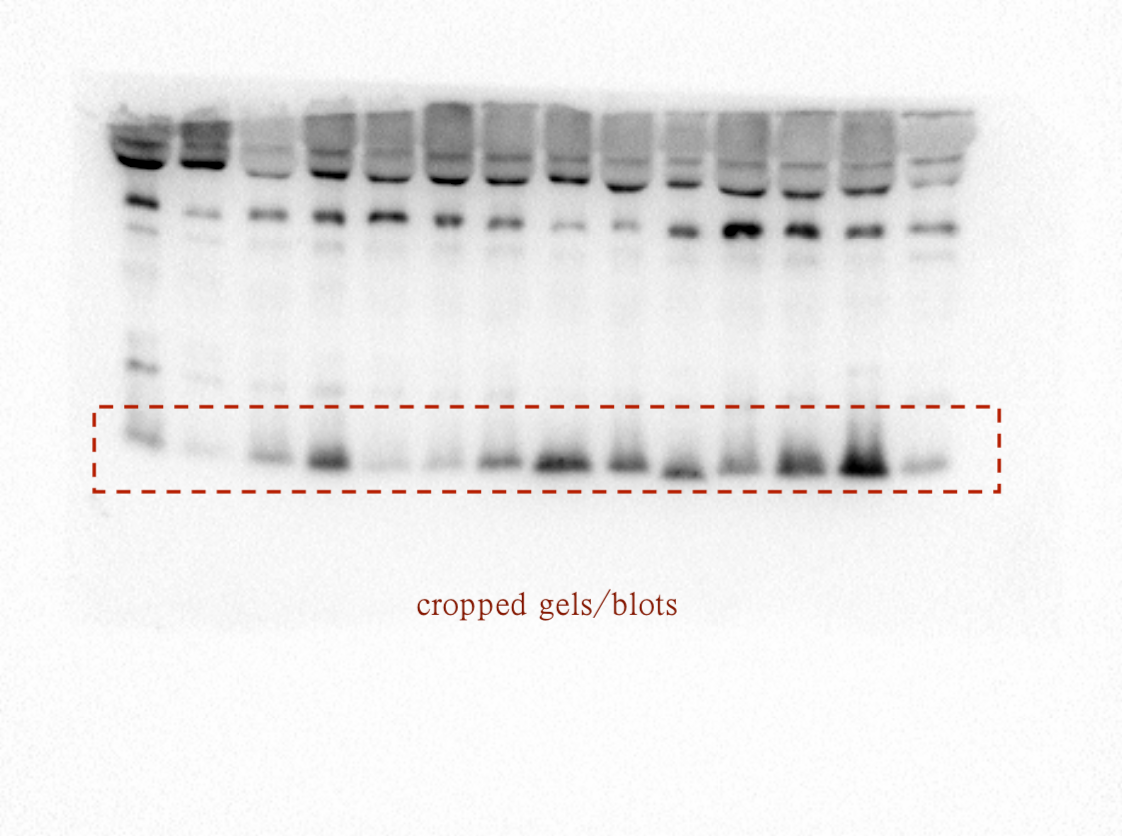

Supplement: Supplementary file 1 — Supplementary Information 1. [file 41598_2022_10936_MOESM1_ESM.tif]

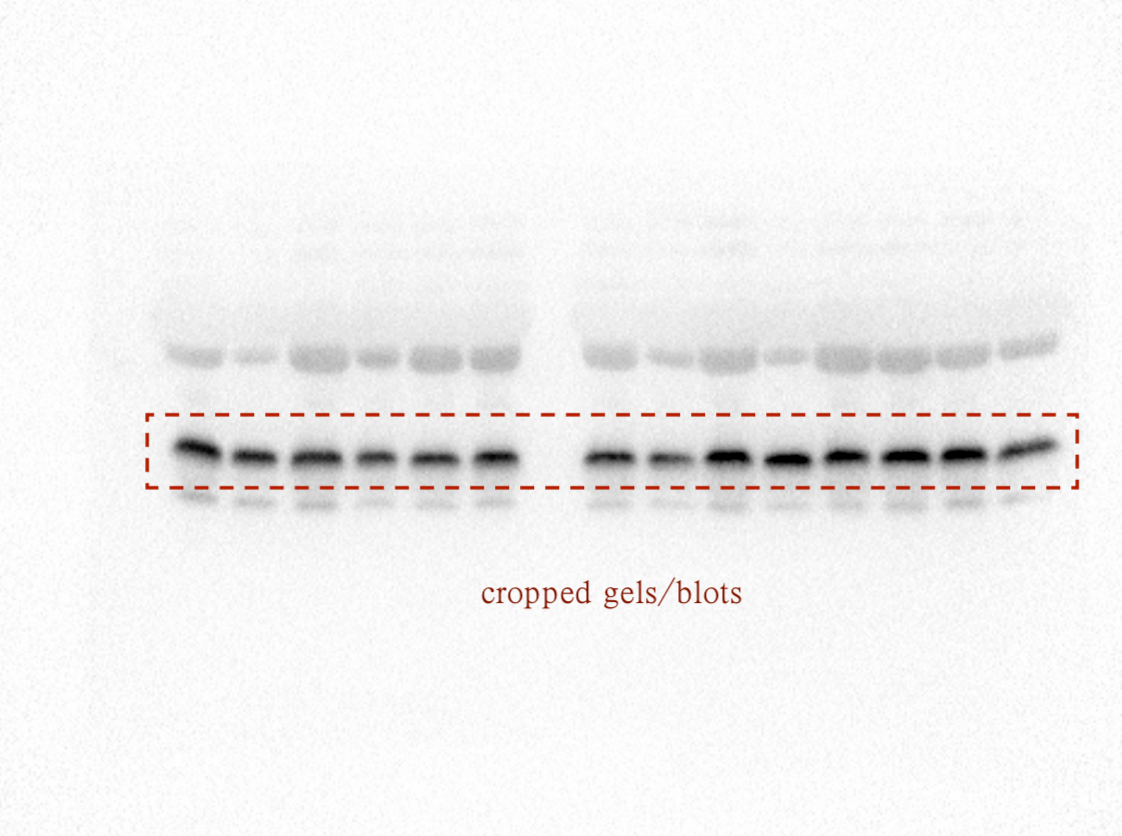

Supplement: Supplementary file 2 — Supplementary Information 2. [file 41598_2022_10936_MOESM2_ESM.tif]
