# Supplementary material for: Brief report on the relation between complement C3a and anti dsDNA antibody in systemic lupus erythematosus
Source: Sci Rep. 2022 May 2;12:7098. doi: 10.1038/s41598-022-10936-z (PMC9061720; doi:10.1038/s41598-022-10936-z)
Supplement: Supplementary file 3 — Supplementary Information 3. [file 41598_2022_10936_MOESM3_ESM.docx]

**supplementary information**

1. (b)

**Figure 1** Correlations between serum C3, SLEDAI scores, and C3a. (a) Reduced serum C3 levels were correlated with SLEDAI scores to some extend. (b) Serum C3a levels exhibited a trend towards correlation with reduced C3. P < 0.05 was the threshold of significance. SLEDAI: Systemic Lupus Erythematosus Disease Activity Index.
